# Some Remarks on Metal Plates

**Published:** 1869-04

**Authors:** B. Wood


### SOME REMARKS ON METAL PLATES.

Since there is a disposition to return to metal plates, I would like to make one or two inquiries and to direct attention to one or two points in relation to them.

Some years ago platina was used in Cincinnati for alloying silver plates, but seems to have been dispensed with. What was the objection? Did it render the plates brittle or impair the color, or was it expected that the small amount of platina used would prevent the plate from becoming black, and was it abandoned because it did not fulfill this (unreasonable) expectation?

It is well known that the alloy of copper in silver or gold, to the amount used in coin or less, will cause these metals to blacken under heat, in consequence of the oxidation of the small quantity of copper on the surface. This is cleaned off by acid. Pure silver and pure gold will not so tarnish under heat. Neither will gold alloyed only with silver, or silver alloyed only with gold. Nor again, if alloyed with platina, or platina and either of the other metals.

I have recently alloyed pure silver for plates, with gold and platina sufficient to give it the requisite hardness and elasticity. It makes a very clean, white plate, that works nicely. It does not blacken under heat. Will it be as liable to tarnish in the mouth as if alloyed with copper? I am aware that the tarnish in the mouth is generally due to the sulphide of silver, but where sulpher is not present in the mouth, I think the plate will keep cleaner.

As to the cracking or splitting of metal plates through the center, as sometimes happens after wearing, a little circumstance lately seemed to afford some explanation of this. I poured some melted silver in a closed crucible, with a view of having the ingot milled out for plate. But, finding deep fissures and grooves in it, I concluded it would not answer, and requested the man who does my milling to melt it over. But, on calling for the plate, I found he had milled the piece just as it was, and, to my surprise, the milling had effectually closed up all the fissures and grooves. But, on straining and bending it, I found it disposed to crack along these places. It occurred to me that plates would be less liable to give trouble afterward if we were always careful to see that the ingots, before milling, were without cracks or flaws. What is your experience? Yours truly,

B. Wood.

